# Supplementary material for: Resolving the full spectrum of human genome variation using Linked-Reads
Source: Genome Res. 2019 Apr;29(4):635–45. doi: 10.1101/gr.234443.118 (PMC6442396; doi:10.1101/gr.234443.118)
Supplement: Supplemental Material [file supp_gr.234443.118_Supplemental_longranger-2.2.zip › longranger-2.2.tar.bz2!/lr2.2-public/lib/stan/cmdstan/stan/doc/api/html/neg__binomial_8hpp_source.html]

Stan: src/stan/prob/distributions/univariate/discrete/neg\_binomial.hpp Source File


|  |  |
| --- | --- |
|  | Stan  2.5.0  probability, sampling & optimization |


- Main Page
- Related Pages
- Namespaces
- Classes
- Files

- File List
- File Members

All Classes Namespaces Files Functions Variables Typedefs Enumerations Enumerator Friends Macros Pages

- src
- stan
- prob
- distributions
- univariate
- discrete

neg\_binomial.hpp

Go to the documentation of this file.

1 #ifndef STAN\_\_PROB\_\_DISTRIBUTIONS\_\_UNIVARIATE\_\_DISCRETE\_\_NEG\_BINOMIAL\_HPP

2 #define STAN\_\_PROB\_\_DISTRIBUTIONS\_\_UNIVARIATE\_\_DISCRETE\_\_NEG\_BINOMIAL\_HPP

3

4 #include <boost/random/negative\_binomial\_distribution.hpp>

5 #include <boost/random/variate\_generator.hpp>

6

7 #include <boost/math/special\_functions/digamma.hpp>

8 #include <stan/agrad/partials\_vari.hpp>

9 #include <stan/math/error\_handling.hpp>

10 #include <stan/math/constants.hpp>

11 #include <stan/math/functions/multiply\_log.hpp>

12 #include <stan/math/functions/value\_of.hpp>

13 #include <stan/meta/traits.hpp>

14 #include <stan/prob/traits.hpp>

15 #include <stan/prob/constants.hpp>

16 #include <stan/prob/internal\_math.hpp>

17 #include <stan/prob/distributions/univariate/continuous/gamma.hpp>

18 #include <stan/prob/distributions/univariate/discrete/poisson.hpp>

19 #include <stan/math/functions/binomial\_coefficient\_log.hpp>

20

21 namespace stan {

22

23  namespace prob {

24

25  // NegBinomial(n|alpha,beta) [alpha > 0; beta > 0; n >= 0]

26  template <bool propto,

27  typename T\_n,

28  typename T\_shape, typename T\_inv\_scale>

29  typename return\_type<T\_shape, T\_inv\_scale>::type

30  neg\_binomial\_log(const T\_n& n,

31  const T\_shape& alpha,

32  const T\_inv\_scale& beta) {

33

34  static const char\* function = "stan::prob::neg\_binomial\_log(%1%)";

35

36  using stan::math::check\_positive\_finite;

37  using stan::math::check\_nonnegative;

38  using stan::math::value\_of;

39  using stan::math::check\_consistent\_sizes;

40  using stan::prob::include\_summand;

41

42  // check if any vectors are zero length

43  if (!(stan::length(n)

44  && stan::length(alpha)

45  && stan::length(beta)))

46  return 0.0;

47

48  double logp(0.0);

49  check\_nonnegative(function, n, "Failures variable", &logp);

50  check\_positive\_finite(function, alpha, "Shape parameter", &logp);

51  check\_positive\_finite(function, beta, "Inverse scale parameter", &logp);

52  check\_consistent\_sizes(function,

53  n,alpha,beta,

54  "Failures variable",

55  "Shape parameter","Inverse scale parameter",

56  &logp);

57

58  // check if no variables are involved and prop-to

59  if (!include\_summand<propto,T\_shape,T\_inv\_scale>::value)

60  return 0.0;

61

62  using stan::math::multiply\_log;

63  using stan::math::binomial\_coefficient\_log;

64  using boost::math::digamma;

65  using boost::math::lgamma;

66

67  // set up template expressions wrapping scalars into vector views

68  VectorView<const T\_n> n\_vec(n);

69  VectorView<const T\_shape> alpha\_vec(alpha);

70  VectorView<const T\_inv\_scale> beta\_vec(beta);

71  size\_t size = max\_size(n, alpha, beta);

72

73  agrad::OperandsAndPartials<T\_shape,T\_inv\_scale>

74  operands\_and\_partials(alpha,beta);

75

76  size\_t len\_ab = max\_size(alpha,beta);

77  DoubleVectorView<true,(is\_vector<T\_shape>::value

78  || is\_vector<T\_inv\_scale>::value)>

79  lambda(len\_ab);

80  for (size\_t i = 0; i < len\_ab; ++i)

81  lambda[i] = value\_of(alpha\_vec[i]) / value\_of(beta\_vec[i]);

82

83  DoubleVectorView<true,is\_vector<T\_inv\_scale>::value>

84  log1p\_beta(length(beta));

85  for (size\_t i = 0; i < length(beta); ++i)

86  log1p\_beta[i] = log1p(value\_of(beta\_vec[i]));

87

88  DoubleVectorView<true,is\_vector<T\_inv\_scale>::value>

89  log\_beta\_m\_log1p\_beta(length(beta));

90  for (size\_t i = 0; i < length(beta); ++i)

91  log\_beta\_m\_log1p\_beta[i] = log(value\_of(beta\_vec[i])) - log1p\_beta[i];

92

93  DoubleVectorView<true,(is\_vector<T\_inv\_scale>::value

94  || is\_vector<T\_shape>::value)>

95  alpha\_times\_log\_beta\_over\_1p\_beta(len\_ab);

96  for (size\_t i = 0; i < len\_ab; ++i)

97  alpha\_times\_log\_beta\_over\_1p\_beta[i]

98  = value\_of(alpha\_vec[i])

99  \* log(value\_of(beta\_vec[i])

100  / (1.0 + value\_of(beta\_vec[i])));

101

102  DoubleVectorView<!is\_constant\_struct<T\_shape>::value,

103  is\_vector<T\_shape>::value>

104  digamma\_alpha(length(alpha));

105  if (!is\_constant\_struct<T\_shape>::value)

106  for (size\_t i = 0; i < length(alpha); ++i)

107  digamma\_alpha[i] = digamma(value\_of(alpha\_vec[i]));

108

109  DoubleVectorView<!is\_constant\_struct<T\_shape>::value,

110  is\_vector<T\_inv\_scale>::value>

111  log\_beta(length(beta));

112  if (!is\_constant\_struct<T\_shape>::value)

113  for (size\_t i = 0; i < length(beta); ++i)

114  log\_beta[i] = log(value\_of(beta\_vec[i]));

115

116  DoubleVectorView<!is\_constant\_struct<T\_inv\_scale>::value,

117  (is\_vector<T\_shape>::value

118  || is\_vector<T\_inv\_scale>::value)>

119  lambda\_m\_alpha\_over\_1p\_beta(len\_ab);

120  if (!is\_constant\_struct<T\_inv\_scale>::value)

121  for (size\_t i = 0; i < len\_ab; ++i)

122  lambda\_m\_alpha\_over\_1p\_beta[i] =

123  lambda[i]

124  - ( value\_of(alpha\_vec[i])

125  / (1.0 + value\_of(beta\_vec[i])) );

126

127  for (size\_t i = 0; i < size; i++) {

128  if (alpha\_vec[i] > 1e10) { // reduces numerically to Poisson

129  if (include\_summand<propto>::value)

130  logp -= lgamma(n\_vec[i] + 1.0);

131  if (include\_summand<propto,T\_shape,T\_inv\_scale>::value)

132  logp += multiply\_log(n\_vec[i], lambda[i]) - lambda[i];

133

134  if (!is\_constant\_struct<T\_shape>::value)

135  operands\_and\_partials.d\_x1[i]

136  += n\_vec[i] / value\_of(alpha\_vec[i])

137  - 1.0 / value\_of(beta\_vec[i]);

138  if (!is\_constant\_struct<T\_inv\_scale>::value)

139  operands\_and\_partials.d\_x2[i]

140  += (lambda[i] - n\_vec[i]) / value\_of(beta\_vec[i]) ;

141  } else { // standard density definition

142  if (include\_summand<propto,T\_shape>::value)

143  if (n\_vec[i] != 0)

144  logp += binomial\_coefficient\_log<double>(n\_vec[i]

145  + value\_of(alpha\_vec[i])

146  - 1.0,

147  n\_vec[i]);

148  if (include\_summand<propto,T\_shape,T\_inv\_scale>::value)

149  logp +=

150  alpha\_times\_log\_beta\_over\_1p\_beta[i]

151  - n\_vec[i] \* log1p\_beta[i];

152

153  if (!is\_constant\_struct<T\_shape>::value)

154  operands\_and\_partials.d\_x1[i]

155  += digamma(value\_of(alpha\_vec[i]) + n\_vec[i])

156  - digamma\_alpha[i]

157  + log\_beta\_m\_log1p\_beta[i];

158  if (!is\_constant\_struct<T\_inv\_scale>::value)

159  operands\_and\_partials.d\_x2[i]

160  += lambda\_m\_alpha\_over\_1p\_beta[i]

161  - n\_vec[i] / (value\_of(beta\_vec[i]) + 1.0);

162  }

163  }

164  return operands\_and\_partials.to\_var(logp);

165  }

166

167  template <typename T\_n,

168  typename T\_shape, typename T\_inv\_scale>

169  inline

170  typename return\_type<T\_shape, T\_inv\_scale>::type

171  neg\_binomial\_log(const T\_n& n,

172  const T\_shape& alpha,

173  const T\_inv\_scale& beta) {

174  return neg\_binomial\_log<false>(n,alpha,beta);

175  }

176

177  // Negative Binomial CDF

178  template <typename T\_n, typename T\_shape,

179  typename T\_inv\_scale>

180  typename return\_type<T\_shape, T\_inv\_scale>::type

181  neg\_binomial\_cdf(const T\_n& n, const T\_shape& alpha,

182  const T\_inv\_scale& beta) {

183  static const char\* function = "stan::prob::neg\_binomial\_cdf(%1%)";

184

185  using stan::math::check\_positive\_finite;

186  using stan::math::check\_nonnegative;

187  using stan::math::check\_consistent\_sizes;

188  using stan::prob::include\_summand;

189

190  // Ensure non-zero arugment lengths

191  if (!(stan::length(n) && stan::length(alpha) && stan::length(beta)))

192  return 1.0;

193

194  double P(1.0);

195

196  // Validate arguments

197  check\_positive\_finite(function, alpha, "Shape parameter", &P);

198  check\_positive\_finite(function, beta, "Inverse scale parameter", &P);

199  check\_consistent\_sizes(function,

200  n, alpha, beta,

201  "Failures variable",

202  "Shape parameter",

203  "Inverse scale parameter",

204  &P);

205

206  // Wrap arguments in vector views

207  VectorView<const T\_n> n\_vec(n);

208  VectorView<const T\_shape> alpha\_vec(alpha);

209  VectorView<const T\_inv\_scale> beta\_vec(beta);

210  size\_t size = max\_size(n, alpha, beta);

211

212  // Compute vectorized CDF and gradient

213  using stan::math::value\_of;

214  using boost::math::ibeta;

215  using boost::math::ibeta\_derivative;

216

217  using boost::math::digamma;

218

219  agrad::OperandsAndPartials<T\_shape, T\_inv\_scale>

220  operands\_and\_partials(alpha, beta);

221

222  // Explicit return for extreme values

223  // The gradients are technically ill-defined, but treated as zero

224  for (size\_t i = 0; i < stan::length(n); i++) {

225  if (value\_of(n\_vec[i]) <= 0)

226  return operands\_and\_partials.to\_var(0.0);

227  }

228

229  // Cache a few expensive function calls if alpha is a parameter

230  DoubleVectorView<!is\_constant\_struct<T\_shape>::value,

231  is\_vector<T\_shape>::value>

232  digammaN\_vec(stan::length(alpha));

233  DoubleVectorView<!is\_constant\_struct<T\_shape>::value,

234  is\_vector<T\_shape>::value>

235  digammaAlpha\_vec(stan::length(alpha));

236  DoubleVectorView<!is\_constant\_struct<T\_shape>::value,

237  is\_vector<T\_shape>::value>

238  digammaSum\_vec(stan::length(alpha));

239  DoubleVectorView<!is\_constant\_struct<T\_shape>::value,

240  is\_vector<T\_shape>::value>

241  betaFunc\_vec(stan::length(alpha));

242

243  if (!is\_constant\_struct<T\_shape>::value) {

244

245  for (size\_t i = 0; i < stan::length(alpha); i++) {

246  const double n\_dbl = value\_of(n\_vec[i]);

247  const double alpha\_dbl = value\_of(alpha\_vec[i]);

248

249  digammaN\_vec[i] = digamma(n\_dbl + 1);

250  digammaAlpha\_vec[i] = digamma(alpha\_dbl);

251  digammaSum\_vec[i] = digamma(n\_dbl + alpha\_dbl + 1);

252  betaFunc\_vec[i] = boost::math::beta(n\_dbl + 1, alpha\_dbl);

253  }

254  }

255

256  for (size\_t i = 0; i < size; i++) {

257

258  // Explicit results for extreme values

259  // The gradients are technically ill-defined, but treated as zero

260  if (value\_of(n\_vec[i])

261  == std::numeric\_limits<double>::infinity())

262  continue;

263

264  const double n\_dbl = value\_of(n\_vec[i]);

265  const double alpha\_dbl = value\_of(alpha\_vec[i]);

266  const double beta\_dbl = value\_of(beta\_vec[i]);

267

268  const double p\_dbl = beta\_dbl / (1.0 + beta\_dbl);

269  const double d\_dbl = 1.0 / ( (1.0 + beta\_dbl)

270  \* (1.0 + beta\_dbl) );

271

272  const double Pi = ibeta(alpha\_dbl, n\_dbl + 1.0, p\_dbl);

273

274  P \*= Pi;

275

276  if (!is\_constant\_struct<T\_shape>::value) {

277

278  double g1 = 0;

279  double g2 = 0;

280

281  stan::math::gradRegIncBeta(g1, g2, alpha\_dbl,

282  n\_dbl + 1, p\_dbl,

283  digammaAlpha\_vec[i],

284  digammaN\_vec[i],

285  digammaSum\_vec[i],

286  betaFunc\_vec[i]);

287

288  operands\_and\_partials.d\_x1[i]

289  += g1 / Pi;

290  }

291

292  if (!is\_constant\_struct<T\_inv\_scale>::value)

293  operands\_and\_partials.d\_x2[i]

294  += d\_dbl \* ibeta\_derivative(alpha\_dbl, n\_dbl + 1, p\_dbl)

295  / Pi;

296

297  }

298

299  if (!is\_constant\_struct<T\_shape>::value)

300  for(size\_t i = 0; i < stan::length(alpha); ++i)

301  operands\_and\_partials.d\_x1[i] \*= P;

302

303  if (!is\_constant\_struct<T\_inv\_scale>::value)

304  for(size\_t i = 0; i < stan::length(beta); ++i)

305  operands\_and\_partials.d\_x2[i] \*= P;

306

307  return operands\_and\_partials.to\_var(P);

308

309  }

310

311  template <typename T\_n, typename T\_shape,

312  typename T\_inv\_scale>

313  typename return\_type<T\_shape, T\_inv\_scale>::type

314  neg\_binomial\_cdf\_log(const T\_n& n, const T\_shape& alpha,

315  const T\_inv\_scale& beta) {

316  static const char\* function = "stan::prob::neg\_binomial\_cdf\_log(%1%)";

317

318  using stan::math::check\_positive\_finite;

319  using stan::math::check\_nonnegative;

320  using stan::math::check\_consistent\_sizes;

321  using stan::prob::include\_summand;

322

323  // Ensure non-zero arugment lengths

324  if (!(stan::length(n) && stan::length(alpha) && stan::length(beta)))

325  return 0.0;

326

327  double P(0.0);

328

329  // Validate arguments

330  check\_positive\_finite(function, alpha, "Shape parameter", &P);

331  check\_positive\_finite(function, beta, "Inverse scale parameter", &P);

332  check\_consistent\_sizes(function,

333  n, alpha, beta,

334  "Failures variable",

335  "Shape parameter",

336  "Inverse scale parameter",

337  &P);

338

339  // Wrap arguments in vector views

340  VectorView<const T\_n> n\_vec(n);

341  VectorView<const T\_shape> alpha\_vec(alpha);

342  VectorView<const T\_inv\_scale> beta\_vec(beta);

343  size\_t size = max\_size(n, alpha, beta);

344

345  // Compute vectorized cdf\_log and gradient

346  using stan::math::value\_of;

347  using boost::math::ibeta;

348  using boost::math::ibeta\_derivative;

349

350  using boost::math::digamma;

351

352  agrad::OperandsAndPartials<T\_shape, T\_inv\_scale>

353  operands\_and\_partials(alpha, beta);

354

355  // Explicit return for extreme values

356  // The gradients are technically ill-defined, but treated as zero

357  for (size\_t i = 0; i < stan::length(n); i++) {

358  if (value\_of(n\_vec[i]) <= 0)

359  return operands\_and\_partials.to\_var(stan::math::negative\_infinity());

360  }

361

362  // Cache a few expensive function calls if alpha is a parameter

363  DoubleVectorView<!is\_constant\_struct<T\_shape>::value,

364  is\_vector<T\_shape>::value>

365  digammaN\_vec(stan::length(alpha));

366  DoubleVectorView<!is\_constant\_struct<T\_shape>::value,

367  is\_vector<T\_shape>::value>

368  digammaAlpha\_vec(stan::length(alpha));

369  DoubleVectorView<!is\_constant\_struct<T\_shape>::value,

370  is\_vector<T\_shape>::value>

371  digammaSum\_vec(stan::length(alpha));

372  DoubleVectorView<!is\_constant\_struct<T\_shape>::value,

373  is\_vector<T\_shape>::value>

374  betaFunc\_vec(stan::length(alpha));

375

376  if (!is\_constant\_struct<T\_shape>::value) {

377  for (size\_t i = 0; i < stan::length(alpha); i++) {

378  const double n\_dbl = value\_of(n\_vec[i]);

379  const double alpha\_dbl = value\_of(alpha\_vec[i]);

380

381  digammaN\_vec[i] = digamma(n\_dbl + 1);

382  digammaAlpha\_vec[i] = digamma(alpha\_dbl);

383  digammaSum\_vec[i] = digamma(n\_dbl + alpha\_dbl + 1);

384  betaFunc\_vec[i] = boost::math::beta(n\_dbl + 1, alpha\_dbl);

385  }

386  }

387

388  for (size\_t i = 0; i < size; i++) {

389  // Explicit results for extreme values

390  // The gradients are technically ill-defined, but treated as zero

391  if (value\_of(n\_vec[i])

392  == std::numeric\_limits<double>::infinity())

393  continue;

394

395  const double n\_dbl = value\_of(n\_vec[i]);

396  const double alpha\_dbl = value\_of(alpha\_vec[i]);

397  const double beta\_dbl = value\_of(beta\_vec[i]);

398  const double p\_dbl = beta\_dbl / (1.0 + beta\_dbl);

399  const double d\_dbl = 1.0 / ( (1.0 + beta\_dbl)

400  \* (1.0 + beta\_dbl) );

401  const double Pi = ibeta(alpha\_dbl, n\_dbl + 1.0, p\_dbl);

402

403  P += log(Pi);

404

405  if (!is\_constant\_struct<T\_shape>::value) {

406  double g1 = 0;

407  double g2 = 0;

408

409  stan::math::gradRegIncBeta(g1, g2, alpha\_dbl,

410  n\_dbl + 1, p\_dbl,

411  digammaAlpha\_vec[i],

412  digammaN\_vec[i],

413  digammaSum\_vec[i],

414  betaFunc\_vec[i]);

415  operands\_and\_partials.d\_x1[i] += g1 / Pi;

416  }

417  if (!is\_constant\_struct<T\_inv\_scale>::value)

418  operands\_and\_partials.d\_x2[i]

419  += d\_dbl \* ibeta\_derivative(alpha\_dbl, n\_dbl + 1, p\_dbl) / Pi;

420  }

421

422  return operands\_and\_partials.to\_var(P);

423  }

424

425  template <typename T\_n, typename T\_shape,

426  typename T\_inv\_scale>

427  typename return\_type<T\_shape, T\_inv\_scale>::type

428  neg\_binomial\_ccdf\_log(const T\_n& n, const T\_shape& alpha,

429  const T\_inv\_scale& beta) {

430  static const char\* function = "stan::prob::neg\_binomial\_ccdf\_log(%1%)";

431

432  using stan::math::check\_positive\_finite;

433  using stan::math::check\_nonnegative;

434  using stan::math::check\_consistent\_sizes;

435  using stan::prob::include\_summand;

436

437  // Ensure non-zero arugment lengths

438  if (!(stan::length(n) && stan::length(alpha) && stan::length(beta)))

439  return 0.0;

440

441  double P(0.0);

442

443  // Validate arguments

444  check\_positive\_finite(function, alpha, "Shape parameter", &P);

445  check\_positive\_finite(function, beta, "Inverse scale parameter", &P);

446  check\_consistent\_sizes(function,

447  n, alpha, beta,

448  "Failures variable",

449  "Shape parameter",

450  "Inverse scale parameter",

451  &P);

452

453  // Wrap arguments in vector views

454  VectorView<const T\_n> n\_vec(n);

455  VectorView<const T\_shape> alpha\_vec(alpha);

456  VectorView<const T\_inv\_scale> beta\_vec(beta);

457  size\_t size = max\_size(n, alpha, beta);

458

459  // Compute vectorized cdf\_log and gradient

460  using stan::math::value\_of;

461  using boost::math::ibeta;

462  using boost::math::ibeta\_derivative;

463

464  using boost::math::digamma;

465

466  agrad::OperandsAndPartials<T\_shape, T\_inv\_scale>

467  operands\_and\_partials(alpha, beta);

468

469  // Explicit return for extreme values

470  // The gradients are technically ill-defined, but treated as zero

471  for (size\_t i = 0; i < stan::length(n); i++) {

472  if (value\_of(n\_vec[i]) <= 0)

473  return operands\_and\_partials.to\_var(0.0);

474  }

475

476  // Cache a few expensive function calls if alpha is a parameter

477  DoubleVectorView<!is\_constant\_struct<T\_shape>::value,

478  is\_vector<T\_shape>::value>

479  digammaN\_vec(stan::length(alpha));

480  DoubleVectorView<!is\_constant\_struct<T\_shape>::value,

481  is\_vector<T\_shape>::value>

482  digammaAlpha\_vec(stan::length(alpha));

483  DoubleVectorView<!is\_constant\_struct<T\_shape>::value,

484  is\_vector<T\_shape>::value>

485  digammaSum\_vec(stan::length(alpha));

486  DoubleVectorView<!is\_constant\_struct<T\_shape>::value,

487  is\_vector<T\_shape>::value>

488  betaFunc\_vec(stan::length(alpha));

489

490  if (!is\_constant\_struct<T\_shape>::value) {

491  for (size\_t i = 0; i < stan::length(alpha); i++) {

492  const double n\_dbl = value\_of(n\_vec[i]);

493  const double alpha\_dbl = value\_of(alpha\_vec[i]);

494

495  digammaN\_vec[i] = digamma(n\_dbl + 1);

496  digammaAlpha\_vec[i] = digamma(alpha\_dbl);

497  digammaSum\_vec[i] = digamma(n\_dbl + alpha\_dbl + 1);

498  betaFunc\_vec[i] = boost::math::beta(n\_dbl + 1, alpha\_dbl);

499  }

500  }

501

502  for (size\_t i = 0; i < size; i++) {

503  // Explicit results for extreme values

504  // The gradients are technically ill-defined, but treated as zero

505  if (value\_of(n\_vec[i])

506  == std::numeric\_limits<double>::infinity())

507  return operands\_and\_partials.to\_var(stan::math::negative\_infinity());

508

509  const double n\_dbl = value\_of(n\_vec[i]);

510  const double alpha\_dbl = value\_of(alpha\_vec[i]);

511  const double beta\_dbl = value\_of(beta\_vec[i]);

512  const double p\_dbl = beta\_dbl / (1.0 + beta\_dbl);

513  const double d\_dbl = 1.0 / ( (1.0 + beta\_dbl)

514  \* (1.0 + beta\_dbl) );

515  const double Pi = 1.0 - ibeta(alpha\_dbl, n\_dbl + 1.0, p\_dbl);

516

517  P += log(Pi);

518

519  if (!is\_constant\_struct<T\_shape>::value) {

520  double g1 = 0;

521  double g2 = 0;

522

523  stan::math::gradRegIncBeta(g1, g2, alpha\_dbl,

524  n\_dbl + 1, p\_dbl,

525  digammaAlpha\_vec[i],

526  digammaN\_vec[i],

527  digammaSum\_vec[i],

528  betaFunc\_vec[i]);

529  operands\_and\_partials.d\_x1[i] -= g1 / Pi;

530  }

531  if (!is\_constant\_struct<T\_inv\_scale>::value)

532  operands\_and\_partials.d\_x2[i]

533  -= d\_dbl \* ibeta\_derivative(alpha\_dbl, n\_dbl + 1, p\_dbl) / Pi;

534  }

535

536  return operands\_and\_partials.to\_var(P);

537  }

538

539  template <class RNG>

540  inline int

541  neg\_binomial\_rng(const double alpha,

542  const double beta,

543  RNG& rng) {

544  using boost::variate\_generator;

545  using boost::random::negative\_binomial\_distribution;

546

547  static const char\* function = "stan::prob::neg\_binomial\_rng(%1%)";

548

549  using stan::math::check\_positive\_finite;

550

551  check\_positive\_finite(function, alpha, "Shape parameter", (double\*)0);

552  check\_positive\_finite(function, beta, "Inverse scale parameter",

553  (double\*)0);

554

555  return stan::prob::poisson\_rng(stan::prob::gamma\_rng(alpha, beta,

556  rng),rng);

557  }

558  }

559 }

560 #endif

multiply\_log.hpp

stan::math::gradRegIncBeta

void gradRegIncBeta(double &g1, double &g2, double a, double b, double z, double digammaA, double digammaB, double digammaSum, double betaAB)

**Definition:** internal\_math.hpp:161

traits.hpp

stan::agrad::OperandsAndPartials::to\_var

T\_return\_type to\_var(double logp)

**Definition:** partials\_vari.hpp:138

stan::math::check\_positive\_finite

bool check\_positive\_finite(const char \*function, const T\_y &y, const char \*name, T\_result \*result)

**Definition:** check\_positive\_finite.hpp:12

error\_handling.hpp

stan::math::multiply\_log

boost::math::tools::promote\_args< T\_a, T\_b >::type multiply\_log(const T\_a a, const T\_b b)

Calculated the value of the first argument times log of the second argument while behaving properly w...

**Definition:** multiply\_log.hpp:51

poisson.hpp

stan::length

size\_t length(const T &)

**Definition:** traits.hpp:159

stan::math::binomial\_coefficient\_log

boost::math::tools::promote\_args< T\_N, T\_n >::type binomial\_coefficient\_log(const T\_N N, const T\_n n)

Return the log of the binomial coefficient for the specified arguments.

**Definition:** binomial\_coefficient\_log.hpp:63

stan::DoubleVectorView

DoubleVectorView allocates double values to be used as intermediate values.

**Definition:** traits.hpp:358

partials\_vari.hpp

stan::agrad::value\_of

T value\_of(const fvar< T > &v)

Return the value of the specified variable.

**Definition:** value\_of.hpp:16

constants.hpp

stan::is\_vector

**Definition:** traits.hpp:83

stan::agrad::lgamma

fvar< T > lgamma(const fvar< T > &x)

**Definition:** lgamma.hpp:15

stan::agrad::OperandsAndPartials

A variable implementation that stores operands and derivatives with respect to the variable...

**Definition:** partials\_vari.hpp:76

traits.hpp

stan::return\_type::type

boost::math::tools::promote\_args< typename scalar\_type< T1 >::type, typename scalar\_type< T2 >::type, typename scalar\_type< T3 >::type, typename scalar\_type< T4 >::type, typename scalar\_type< T5 >::type, typename scalar\_type< T6 >::type >::type type

**Definition:** traits.hpp:406

stan::is\_constant\_struct

Metaprogram to determine if a type has a base scalar type that can be assigned to type double...

**Definition:** traits.hpp:57

stan::math::value\_of

double value\_of(const T x)

Return the value of the specified scalar argument converted to a double value.

**Definition:** value\_of.hpp:24

stan::prob::include\_summand

Template metaprogram to calculate whether a summand needs to be included in a proportional (log) prob...

**Definition:** traits.hpp:35

stan::prob::poisson\_rng

int poisson\_rng(const double lambda, RNG &rng)

**Definition:** poisson.hpp:384

stan::prob::neg\_binomial\_ccdf\_log

return\_type< T\_shape, T\_inv\_scale >::type neg\_binomial\_ccdf\_log(const T\_n &n, const T\_shape &alpha, const T\_inv\_scale &beta)

**Definition:** neg\_binomial.hpp:428

stan::agrad::OperandsAndPartials::d\_x2

VectorView< double \*, is\_vector< T2 >::value, is\_constant\_struct< T2 >::value > d\_x2

**Definition:** partials\_vari.hpp:83

stan::agrad::ibeta

var ibeta(const var &a, const var &b, const var &x)

The normalized incomplete beta function of a, b, and x.

**Definition:** ibeta.hpp:223

stan::math::check\_nonnegative

bool check\_nonnegative(const char \*function, const T\_y &y, const char \*name, T\_result \*result)

**Definition:** check\_nonnegative.hpp:52

stan::math::check\_consistent\_sizes

bool check\_consistent\_sizes(const char \*function, const T1 &x1, const T2 &x2, const char \*name1, const char \*name2, T\_result \*result)

**Definition:** check\_consistent\_sizes.hpp:13

stan::max\_size

size\_t max\_size(const T1 &x1, const T2 &x2)

**Definition:** traits.hpp:191

internal\_math.hpp

stan::prob::neg\_binomial\_log

return\_type< T\_shape, T\_inv\_scale >::type neg\_binomial\_log(const T\_n &n, const T\_shape &alpha, const T\_inv\_scale &beta)

**Definition:** neg\_binomial.hpp:30

stan::prob::neg\_binomial\_cdf

return\_type< T\_shape, T\_inv\_scale >::type neg\_binomial\_cdf(const T\_n &n, const T\_shape &alpha, const T\_inv\_scale &beta)

**Definition:** neg\_binomial.hpp:181

stan::agrad::multiply\_log

fvar< T > multiply\_log(const fvar< T > &x1, const fvar< T > &x2)

**Definition:** multiply\_log.hpp:15

stan::math::size

int size(const std::vector< T > &x)

**Definition:** size.hpp:11

stan::prob::neg\_binomial\_cdf\_log

return\_type< T\_shape, T\_inv\_scale >::type neg\_binomial\_cdf\_log(const T\_n &n, const T\_shape &alpha, const T\_inv\_scale &beta)

**Definition:** neg\_binomial.hpp:314

stan::agrad::digamma

fvar< T > digamma(const fvar< T > &x)

**Definition:** digamma.hpp:16

value\_of.hpp

stan::agrad::OperandsAndPartials::d\_x1

VectorView< double \*, is\_vector< T1 >::value, is\_constant\_struct< T1 >::value > d\_x1

**Definition:** partials\_vari.hpp:82

binomial\_coefficient\_log.hpp

constants.hpp

stan::prob::neg\_binomial\_rng

int neg\_binomial\_rng(const double alpha, const double beta, RNG &rng)

**Definition:** neg\_binomial.hpp:541

stan::agrad::log

fvar< T > log(const fvar< T > &x)

**Definition:** log.hpp:15

stan::VectorView

VectorView is a template metaprogram that takes its argument and allows it to be used like a vector...

**Definition:** traits.hpp:275

stan::prob::gamma\_rng

double gamma\_rng(const double alpha, const double beta, RNG &rng)

**Definition:** gamma.hpp:491

stan::agrad::log1p

fvar< T > log1p(const fvar< T > &x)

**Definition:** log1p.hpp:16

stan::math::negative\_infinity

double negative\_infinity()

Return negative infinity.

**Definition:** constants.hpp:123

gamma.hpp


---

[ Stan Home Page ]

*© 2011–2014,
Stan Development Team.*
